# Safety of electroconvulsive therapy (ECT) in pregnancy: a systematic review of case reports and case series

**DOI:** 10.1007/s00737-023-01394-1

**Published:** 2023-11-14

**Authors:** Salvatore Cipolla, Pierluigi Catapano, Martin Messina, Pasquale Pezzella, Giulia Maria Giordano

**Affiliations:** Department of Psychiatry, University of Campania “Luigi Vanvitelli”, 80138 Naples, Italy

**Keywords:** Electroconvulsive therapy, Pregnancy, Safety, Women, Mental health

## Abstract

Pregnancy and the immediate postpartum period are considered at high risk for women who have already received a previous psychiatric diagnosis and might represent a stressful event favoring the onset of new psychiatric disorders. The electroconvulsive therapy (ECT) is effective for the treatment of severe, treatment-resistant mental disorders, and it could represent a therapeutic choice for psychiatric conditions during pregnancy. The purpose of this systematic review is to evaluate the safety of ECT during pregnancy and to update the state of the art of its use. An extensive literature search on PubMed, APA PsycInfo, and Scopus databases for relevant articles published from inception to September 2023 has been performed. A final number of 45 articles (34 case reports and 11 case series, for a total of 130 pregnant women) were included in the present review. The limited evidence confirmed that ECT is effective in determining a partial remission of symptoms in women suffering from severe mental disorders, especially in the presence of suicidal ideation or psychosis, during all pregnancy epochs. However, ECT is not free from side effects, although the majority of possible complications were of low- or moderate-grade and not life-threatening for the women. Exposure to pharmacological treatment before or during the ECT or to the anesthetic during ECT might have contributed to the onset of these complications. ECT techniques evolved over years, increasing the degree of its safety, and according to our review it appears to be relatively safe and effective during pregnancy in the majority of cases.

## Background

The use of electroconvulsive therapy (ECT) spread as far as it was ideated by Cerletti and Bini in Italy in 1938 (Endler 1988), and it is still one of the therapeutic options considered for patients affected by many neuropsychiatric disorders, in particular for those who failed to respond to pharmacological treatments (Weiner and Reti 2017; Correll et al. 2021).

The technique is characterized by the use of electric impulses passed through the scalp to induce brain stimulation and consequential therapeutic generalized seizure; nowadays, the modified ECT (MECT) involves the administration of electric impulses under brief anesthesia and muscle relaxants (Volkow 2021; Salinas 2022).

Many studies indicate a good ECT effectiveness, but, despite its decades-long use, the exact mechanism of action still remains unclear (Bolwig 2011; Farzan et al. 2014; Haskett 2014; Nie et al. 2022).

Side effects can derive both directly from the electrical activity induced by ECT and indirectly from the anesthetic drugs used during the treatment (Andrade et al. 2016). The most common side effects reported are localized pain or injury, myalgia, headache, nausea, confusion, and anterograde and retrograde amnesia (usually transient). More rarely severe side effects have been reported including prolonged seizures and status epilepticus, laryngospasm, peripheral nerve palsy, onset of manic/hypomanic symptoms, transient hemiparesis, hemianopsia, dysphasia, arrhythmia, and even death (Kriss et al. 1978; Zavorotnyy et al. 2009; Andrade et al. 2016).

This technique is often used in the context of severe mental disorders (Freedman et al. 2021), such as severe treatment-resistant major depression with severely debilitating symptoms and suicidal ideation (Hermida et al. 2018; Kroenke 2021), treatment resistant schizophrenia (Ali et al. 2019), bipolar disorder (Perugi et al. 2017, 2020), severe catatonia (Pelzer et al. 2018), neuroleptic malignant syndrome (Kuhlwilm et al. 2020), delirium (Lupke et al. 2022), Parkinson’s disease (Takamiya et al. 2021), and autism spectrum disorders (Park et al. 2021; Arango et al. 2021).

Noteworthy is also the use of ECT in pregnant patients suffering from severe mental disorders. Indeed, pregnancy is a unique experience for women that implies biological, psychic, and social changes, and in some cases, it might represent a stressful event that favors the onset of new psychiatric disorders in the postpartum (Soet et al. 2003; Nordentoft et al. 2021; Patton et al. 2021; Galbally et al. 2022). Furthermore, pregnancy and the immediate postpartum period are considered at high risk for women who have already received a previous psychiatric diagnosis, potentially leading to a re-exacerbation of the clinical manifestations (Gentile 2005; Cuijpers et al. 2021; Fusar-Poli et al. 2021; Reichenberg and Levine 2021). A prospective study from Dietz and colleagues showed that, among 4398 enrolled pregnant women, 15.4% had depression identified during at least one pregnancy phase and 17.5% also had a diagnosis of anxiety (Dietz et al. 2007; Furukawa et al. 2021; Stein et al. 2021). As regard to psychotic symptoms, they also may appear suddenly and acutely during pregnancy or in the postpartum period (Rai et al. 2015; Maj et al. 2021; Peralta et al. 2021; Fusar-Poli et al. 2022). Throughout pregnancy, women may suffer from many other psychiatric disorders, such as bipolar disorder, post-partum depression, eating disorders, obsessive–compulsive disorder, posttraumatic stress disorder, panic attacks, and puerperal psychosis (McIntyre et al. 2022; Ortega et al. 2023). However, peripartum mental illnesses are largely under-diagnosed, and often undertreated (Marcus et al. 2003), specially due to the risk of adverse effects of psychiatric drugs on the fetus (harelip, cleft palate, floppy infant, Ebstein’s anomaly, cardiac, lung and kidney alterations), teratogenicity and fetal toxicity, as well as to the possible increase of side effects in the mother (Mesches et al. 2020; Wang et al. 2022; Zheng et al. 2022). Other reasons might include the following: the lack of specialized mental health during pregnancy resources, the poor connection between gynecology and mental health services (World Health Organization 2022), as well as the reluctance of partners and family members to recognize and respond to mothers’ emotional and practical needs (Dennis and Chung-Lee 2006).

Unfortunately, untreated mental illnesses are associated with adverse effects both on the mother and fetus, with severe consequences on maternal and fetus health, which might continue later during childhood and adolescence (Carter et al. 2001). Furthermore, postpartum psychiatric disorders can adversely affect mother-infant dyad (Carter et al. 2001).

In this scenario, ECT would represent a safe and effective therapeutic choice in pregnancy (Pompili et al. 2014). Recently, the Royal College of Psychiatrists stated that ECT is the first-line treatment for pregnant women with severe depression, when there is a serious risk for their physical health or for the fetus, or high suicidal risk, severe psychomotor retardation, and physical deterioration (Waite and Easton 2013; Schomerus et al. 2021).

Unfortunately, ECT suffered from a damaging stigma that has limited its use (Hermida et al. 2018): There is still limited knowledge of this technique and its improvements over time in terms of safety among health professionals and the general population (Wilkinson et al. 2021; Bahji 2022; Salani et al. 2023).

Considering that ECT is an interesting tool for treatment during pregnancy when effective pharmacotherapy cannot be safely implemented, the purpose of this systematic review is to evaluate the safety of ECT during pregnancy and to update the state of the art of its therapeutic use during pregnancy.

## Methods

### Search strategy

An extensive literature search on PubMed, APA PsycInfo, and Scopus databases for relevant articles published from inception to September 2023 has been performed following the Preferred Reporting Items for Systematic Review and Meta-Analysis (PRISMA) guidelines (Liberati et al. 2009). The following search equations were used: ( ( “Electroconvulsive Therapy”[Mesh]) AND ( “Pregnancy”[Mesh])), using “Abstract”, “English”, “Italian”, “Female”, “Humans” as filters on PubMed. On Scopus, the search for terms in the title, abstract and keywords was performed as follows: TITLE-ABS-KEY ( ( “Electroconvulsive Therapy” OR “ECT”) AND ( “Pregnancy” OR “pregnant”)) AND ( LIMIT-TO ( DOCTYPE, “ar”)) AND ( LIMIT-TO ( LANGUAGE, “English”)) AND ( LIMIT-TO ( EXACTKEYWORD, “Human”) OR LIMIT-TO ( EXACTKEYWORD, “Electroconvulsive Therapy”) OR LIMIT-TO ( EXACTKEYWORD, “Pregnancy”)). Finally, the search key on APA PsycInfo was: title((Electroconvulsive Therapy) AND (pregnant OR pregnancy)) AND (rtype.exact(“Journal” NOT (“Letter” OR “Review-Book” OR “Editorial” OR “Column/Opinion” OR “Review-Media” OR “Abstract Collection” OR “Bibliography” OR “Review-Software & Other”)) AND subt.exact((“pregnancy” OR “female” OR “humans”) NOT (“male” OR “surveys and questionnaires” OR “animals” OR “rats” OR “sexual behavior”)) AND la.exact(“ENG”) NOT me.exact(“Interview” OR “Literature Review” OR “Systematic Review” OR “Meta Analysis” OR “Focus Group” OR “Mathematical Model” OR “Nonclinical Case Study” OR “Metasynthesis” OR “Scientific Simulation”) AND po.exact(“Human” NOT (“Male” OR “Animal” OR “Transgender”)) AND PEER(yes)).

### Selection criteria

The following inclusion criteria were used: studies in English involving women undergoing ECT during any trimester of pregnancy. Due to the lack of clinical trials in this specific population, case reports and case series were also included. Systematic review and meta-analyses on the topic were not included in the present systematic review, but they were considered for the interpretation of our results. In addition, reference lists of these systematic review and meta-analyses, as well as those of included articles were screened to identify additional relevant studies.

Furthermore, studies that did not provide adequate information for most of the following variables of interest were excluded: clinical and psychopathological characteristics of the patient, positioning of the electrodes during ECT, number of ECT sessions, occurrence of complications, and clinical outcomes.

### Selection process

Three researchers (SC, PC, and MM) independently screened for eligibility all the articles by titles and abstracts and then proceeded to read the full text. Discrepancies in the selection of the eligible articles were reported to the other authors and were resolved by discussion and consensus.

The papers relating to case reports of individual women have been divided according to the trimester of pregnancy at the time of ECT. The case series reporting information from groups of pregnant women have been summarized in a separate section.

## Results

**Fig. 1:**
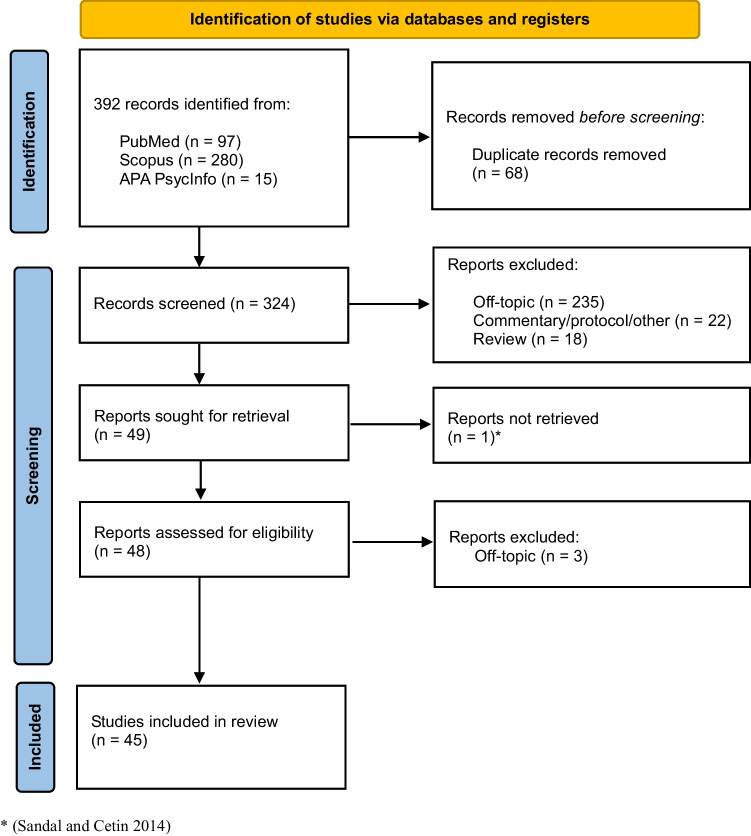
Flowchart of review work

The literature search yielded 392 articles. After removal of duplicated (*N* = 68), 324 records were screened by title and abstract. Of these, 235 papers were excluded because they did not meet the inclusion criteria; 22 were excluded because they were authors’ letters, commentary, guidelines, and protocol articles; and 18 were excluded because they were reviews or meta-analyses. Of the remaining articles, one was excluded because it was not retrieved (Sandal and Cetin 2014), and three were excluded after an in deep full-text reading because it did not meet the eligibility criteria. Thus, a final number of 45 articles were finally included in the present review. Eleven out of 45 were case series; the remaining were single case reports (Fig. 1).

A synthesis of the whole sample’s characteristics is shown in Table 1. A total of 130 cases of pregnant women were considered. The mean age at the time of ECT was 29.6 years (± 6.07) and ranged from 19 years (Laird 1955) to 48 years (Salzbrenner et al. 2011). However, in 60 cases (46.1%), mothers’ age was not specified. As regard to the trimester of pregnancy during which ECT was performed, in 42.3% of cases, it was performed during the second trimester (weeks 12–24), 30% in the third trimester (weeks > 25), 23.8% in the first trimester (weeks 1–12), and in the remaining 3.8% (5 cases out of 130), the trimester of pregnancy was unspecified. In all selected cases, the pregnancy was single except for one case in which a twin pregnancy has been reported (Livingston et al. 1994). In most cases, the main diagnosis for which the pregnant women were advised to practice ECT was major depressive disorder (48.5%), followed by bipolar disorder (24.6% in the total, of which 6.9% with a depressive episode, 7.7% manic episode, 10% unspecified) and schizophrenia spectrum disorders (15.4%); the remaining women had other psychiatric disorders such as panic disorder and obsessive–compulsive disorder (2.3%) or the main diagnosis was not specified (9.2%). In 10 case reports and three case series, a total of 23 comorbidities with the main psychiatric illness were reported: Graves’ disease, hypo- and hyper-thyroidism, diabetes mellitus, gestational diabetes, obesity, arterial hypertension, anemia, cerebral palsy, migraine, hearing loss, ankylosing spondylitis, SARS-CoV-2 and Streptococcus B infections, intellectual disability, post-traumatic stress disorder, obsessive–compulsive disorder, generalized anxiety disorder, panic disorder, conversion disorder, borderline personality disorder, alcohol abuse, and cocaine addiction.

**Table 1:** Summary of the whole sample’s characteristics (*N* = 128)

Trimester of pregnancy at the time of ECT (*n*, %)	
*First trimester* *(1–12 weeks)*	31 (23.8%)
*Second trimester* *(13–24 weeks)*	55 (42.3%)
*Third trimester* *(*> *25 weeks)*	39 (30%)
Unspecified trimester	5 (3.8%)
Principal diagnosis (*n*, %)	
*Major depressive disorder*	63 (48.5%)
*Bipolar disorder — depressive episode*	9 (6.9%)
*Bipolar disorder — manic episode*	10 (7.7%)
*Bipolar disorder — unspecified*	13 (10%)
*Schizophrenia spectrum disorder*	20 (15.4%)
*Other psychiatric disorder*	3 (2.3%)
Unspecified diagnosis	12 (9.2%)
Age at the time of ECT	
*Mean age (DS)*	29.6 (± 6.07)
Unspecified age (*n*, %)	60 (46.1%)

Pharmacological treatment during pregnancy, simultaneously with the execution of ECT, was reported in 24 papers (53.3% of total case reports), with increasing prevalence from first to third trimester. The drugs used belonged to numerous classes including antipsychotics, antidepressants, and hypnotic sedatives with different combinations and dosages.

ECT was performed with bilateral placement of the electrodes in 75 cases (57.7%) and unilateral in 19 cases (14.8%); in 26.9% of cases, electrode placement was unspecified, and in one case (Varan et al. 1985), electrode placement was changed from bilateral to unilateral over the course of sessions. The number of sessions during pregnancy varied, ranging from a minimum of one session (Ray-Griffith et al. 2016) to a maximum of 23 sessions (Rabie et al. 2021). The data on the efficacy of ECT were not always presented in the selected articles: In 82.3%, a partial improvement or a total resolution of the symptoms has been reported; in 13% of the sample, no improvement was reported; and in 4.6%, no information was provided. Regarding the safety of the technique, 38 complications were reported in the mother during or at the end of ECT, while 23 adverse effects were reported in the child. Due to the absence in the literature of a clear classification of the severity of ECT complications, we decided to classify them into three classes: low-, moderate-, and severe-grade complications (from spontaneous resolution with no need of medical intervention to permanent or potentially irreversible damages), as shown in Table 2 and Table 3. In particular, low-grade complications were those that resolved spontaneously and/or did not require medical intervention. These complications included transient changes in heart rate and rhythm, blood pressure, uterine contractions, fetal spasms, pelvic pain, and the appearance of mild symptoms of hypomania. Among these, transient fetal arrythmias and uterine contractions were the most frequent, reported 10 (Varan et al. 1985; Bhatia et al. 1999; DeBattista et al. 2003; Bozkurt et al. 2007; De Asis et al. 2013; Halmo et al. 2014; Moosavi Rineh et al. 2020; Rabie et al. 2021; Bhasin et al. 2022; Patel et al. 2022) and four times (Bhatia et al. 1999; Bulbul et al. 2013; Grover et al. 2017; Rabie et al. 2021), respectively. Moderate-grade complications were those that have not had a rapid resolution and/or have required medical intervention; the most frequent was preterm birth, reported in seven papers (Livingston et al. 1994; Pinette et al. 2007; Kasar et al. 2007; Yang et al. 2011; Bulut et al. 2013; Ray-Griffith et al. 2016; Bhasin et al. 2022). Severe complications are represented by permanent or potentially irreversible damage to the mother and/or the fetus. These include status epilepticus, renal insufficiency, diabetes insipidus, cardiac insufficiency (Balki et al. 2006), prolonged epileptic crisis (De Asis et al. 2013), complete heart block (Ray-Griffith et al. 2016), cerebellar infarct with tonic extension of the upper limbs (Pinette et al. 2007), transient heart failure, congenital hip dysplasia (Bulbul et al 2013), club foot deformity (Bulut et al 2013), and up to child’s death/abortion, reported in five cases (Balki et al. 2006; Bulbul et al. 2013; Livingston et al. 1994; Moreno et al. 1998; Simon 1948). Overall, 50% of maternal complications were moderate, 34.2% low, and 15.8% severe; 47.8% of the complications in the fetus were low, 43.5% severe, and the remaining 8.7% moderate grade (Table 4).

**Table 2:** Classification of ECT-related complications in mother according to the severity

*Low grade (n)*	*Moderate grade (n)*	*Severe grade (n)*
Hypomanic symptoms (3)	Late-onset contraction (1)	Cardiac insufficiency (1)
Pelvic pain (2)	Moderate memory loss (1)	Complete heart block (1)
Supraventricular tachycardia (1)	Placental abruption (1)	Diabetes insipidus (1)
Transient hypotension (1)	Pneumothorax (1)	Prolonged epileptic crisis (1)
Transient increase in hormones (1)	Pre-eclampsia (5)	Renal insufficiency (1)
Transient memory loss (1)	Preterm delivery (7)	Status epilepticus (1)
Uterine contraction (4)	Vaginal bleeding (3)	

In brackets, the number of times that complication has been reported

**Table 3:** Classification of ECT-related complications in child according to the severity

*Low grade (n)*	*Moderate grade (n)*	*Severe grade (n)*
Transient fetal arrhythmias (10)	Hyaline membrane disease (1)	Abortion/death (5)
Transient fetal spasms (1)	Pyloric stenosis (1)	Cerebellar infarct (1)
		Club foot deformity (1)
		Congenital hip dysplasia (1)
		Tonic extension upper limbs (1)
		Transient heart failure (1)

In brackets, the number of times that complication has been reported

**Table 4:** Times ECT-related complications are reported

Complication	*N* (%)
Complications in mothers (*n* = 38)	
*Low grade*	13 (34.2%)
*Moderate grade*	19 (50%)
*Severe grade*	6 (15.8%)
Complications in child (*n* = 23)	
*Low grade*	11 (47.8%)
*Moderate grade*	2 (8.7%)
*Severe grade*	10 (43.5%)

### Case reports

#### First trimester of pregnancy

Four case reports were included in this category (Table 5): three women had a diagnosis of bipolar disorder (one with depressive episode, two with manic episode), and one woman was affected by major depressive disorder. The mean age was 28.2 years. All women reported psychotic symptoms and suicidal ideation, except for one patient who did not present suicidality (Lovas et al. 2011). Two women had organic comorbidities, such as cerebral palsy, hearing loss, obesity, and arterial hypertension. In only one case, haloperidol-based pharmacological treatment was reported together with ECT (Moreno et al. 1998); in the other three reports, no pharmacological treatment was carried out during ECT. In the case report by Dorn (1985), no complications from ECT use were found for either the mother or the child, except for the occurrence of hypomanic symptoms in the mother, classified as low-grade symptoms. In the case report by Moreno et al. (1998), maternal moderate memory loss was reported (moderate-grade complication) and the pregnancy was not completed due to a miscarriage that occurred at the end of the second ECT session at the eighth week of gestation; however, the authors concluded that it was not possible to exclude the role of amitriptyline, haloperidol, and carbamazepine taken in the first weeks of pregnancy, before ECT, in increasing the risk of miscarriage. Ghanizadeh reported only maternal vaginal bleeding (moderate-grade complication) among ECT-related complications (Ghanizadeh et al. 2009). Lovas reported low-grade (pelvic pain) and moderate-grade (pre-eclampsia) complication in mother; however, the latter cannot be definitively related to ECT due to the gravida’s comorbidities (Lovas et al. 2011).

**Table 5:** Case reports on women undergoing electro-convulsant therapy during the first trimester of pregnancy

First author (publication year)	Dx (age)	Mother’s comorbidity				Psychotropic drug(s) taken during ECT	ECT sessions during pregnancy		Symptoms resolution (measure of outcome)	ECT-related complication		Conclusion
		*Catatonic symptoms*	*Psychotic symptoms*	*Suicidality*	*Other*		*n*	*Electrode placement*		*In mother*	*In child*	
Dorn (1985)	BD-D (27)		+	+	Cerebral palsy; hearing loss	None	9	Bilateral	Hypomania (N/A)	Hypomanic symptoms	None	ECT is safe and effective in pregnancy
Moreno et al. (1998)	MDD (25)		** + **	** + **		Haloperidol^*^ 10 mg/day/os	2	Bilateral	Yes (N/A)	Moderate memory loss	Abortion	Prescribing ECT in the first trimester of pregnancy, the risk of spontaneous abortion should be discussed. Other treatments may contribute to the miscarriage
Ghanizadeh et al. (2009)	BD-M (30)		+	+		None	9	N/A	Yes (N/A)	Vaginal bleeding	None	Vaginal bleeding may be a direct effect of ECT, and clinicians should be aware of this complication
Lovas et al. (2011)	BD-M (31)		+		Ob; AH	None	21	Bilateral	Yes (N/A)	Pelvic pain; Pre-eclampsia	None	ECT rapidly minimize psychiatric symptoms and no evidence supports causal relationship with complications in mother

*AH* arterial hypertension, *BD-D* bipolar disorder, depressive episode, *BD-M* bipolar disorder, manic episode, *Dx* principal diagnosis, *ECT* electro-convulsant therapy, *MDD* major depressive disorder, *N/A* not available, *Ob* obesity

^*^First-generation antipsychotic drug

#### Second trimester of pregnancy

Sixteen case reports dealing with women in their second trimester of pregnancy were included (Table 6); the majority of women (10 out of 16) were affected by major depressive disorder, two women (Varan et al. 1985) were affected by schizophrenia and related disorders, and the remaining by bipolar disorder (two with manic episode, one with depressive episode). The average age was 30.7 years, from a minimum of 20 years old (De Asis et al. 2013) to a maximum of 41 years (DeBattista et al. 2003). Four women had catatonic symptoms (Block 1948; Varan et al. 1985; Pinna et al. 2015; Gandhi et al. 2023), seven women psychotic symptoms (Repke and Berger 1984; Varan et al. 1985; Bozkurt et al. 2007; De Asis et al. 2013; Pinna et al. 2015; Bhasin et al. 2022), and six women suicidal ideation (Repke and Berger 1984; Balki et al. 2006; O’Reardon et al. 2011; Gahr et al. 2012; Özgül et al. 2014; Erturk et al. 2020). In one case (O’Reardon et al. 2011), Graves’ disease was reported as comorbidity of major depressive disorder and suicidality; in another case (Bhasin et al. 2022), SARS-CoV-2 infection emerged in the course of pregnancy. Pharmacological treatments based on first- and second-generation antipsychotics, tricyclic antidepressants and selective serotonin reuptake inhibitors, and benzodiazepines have been reported (Table 6). In 13 out of 16 cases, an improvement of symptoms has been reported after treatment with ECT; in the remaining three cases, no information has been provided on the effectiveness of the technique. As regard to complications, low-grade maternal complications included the following: pelvic pain (Bozkurt et al. 2007), hypotension (Repke and Berger 1984), and memory loss (Varan et al. 1985), as well as acute and transient increase in hormonal levels of prolactin, ACTH, norepinephrine, epinephrine, beta-endorphin, dopamine, and oxytocin, in the presence of stable mother’s vital signs (Griffiths et al. 1989).

**Table 6:** Case reports on women undergoing electro-convulsant therapy during the second trimester of pregnancy

First Author (publication year)	Dx (age)	Mother’s comorbidity				Psychotropic drug(s) taken during ECT	ECT sessions during pregnancy		Symptoms resolution (measure of outcome)	ECT-related complication		Conclusion
		*Catatonic symptoms*	*Psychotic symptoms*	*Suicidality*	*Other*		*n*	*Electrode placement*		*In mother*	*In child*	
Block (1948)	MDD (30)	+				None	N/A	N/A	Yes (N/A)	None	None	No complications raised due to ECT treatment
Repke and Berger (1984)	MDD (33)		+	+		Desipramine^†^ 200 mg/day/os	N/A	N/A	Yes (N/A)	Transient hypotension	None	ECT has a minimal effect on the fetus; the mother needs pre-operative hydration
Varan et al. (1985)	SCZ (33)	+	+			CPZ^*^ 600 mg/day/os	12	Bilateral, then right unilateral	Yes (N/A)	Transient memory loss	Transient fetal arrhythmias	The importance of ECT is affirmed, and its relative safety in pregnancy is confirmed
Griffiths et al. (1989)	MDD (30)					N/A	N/A	N/A	N/A	Transient increase in hormones	None	ECT in pregnancy is associated with acute neurohumoral changes, but none of these adversely affects the fetus
DeBattista et al. (2003)	MDD (41)					None	5	Bilateral	Yes (HAM-D)	None	Transient fetal arrhythmias	The clinical significance of fetal hearth rate decelerations during ECT would appear minimal, as these decelerations are brief
Balki et al. (2006)	MDD (31)			+		Paroxetine^††^; Lorazepam^¶^	1	Unilateral	N/A	Status epilepticus; diabetes insipidus; renal insufficiency; cardiac insufficiency	Death	ECT-induced status epilepticus may cause fetal death and major consequences in mother
Bozkurt et al. (2007)	MDD (34)		+			None	22	Bilateral	Yes (HAM-D)	Pelvic pain	Transient fetal arrhythmias	Acute and maintenance ECT may be the choice of treatment in severely depressed or psychotic pregnant patients
Pinette et al. (2007)	MDD (22)					None	7	Bilateral	N/A	Pre-eclampsia; preterm delivery	Cerebellar infarct; tonic extension upper limbs	Uterine contractions and fetal heart rate reduction could lead to fetal compromise
O’Reardon et al. (2011)	MDD (39)			+	Graves’ disease	None	18	Bilateral	Yes (HAM-D; BDI; BAI)	PNX	None	ECT could be a treatment of choice in pregnant women with recurrent and resistant depression and severe symptoms
Gahr et al. (2012)	MDD (35)			+		Fluoxetine^††^ 20 mg/day/os	15	Unilateral	Yes (BDI)	None	None	ECT was effective and well tolerated by patient and fetus. During first trimester, rTMS was performed with no success
De Asis et al. (2013)	BD-D (20)		+			None	14	Unilateral	Yes (N/A)	Prolonged epileptic crisis	Transient fetal arrhythmias	Propofol in pregnant women undergoing ECT may reduce seizure duration and fetal arrhythmias
Özgül et al. (2014)	MDD (32)			+		N/A	10	Bilateral	Yes (N/A)	N/A	N/A	ECT can be provided safely using propofol and succinylcholine as anesthetic agents
Pinna et al. (2015)	BD-M (28)	+	+			Lorazepam^¶^ 30 mg/day/os	9	Bilateral	Yes (N/A)	None	None	ECT is a safe option that allows to breastfeed after delivery as it does not require pharmacological maintenance therapy
Erturk et al. (2020)	MDD (31)			+		Sertraline^††^ 100 mg/day/os	10	Bilateral	Yes (N/A)	None	None	ECT seems to be safe and effective for pregnant women with MDD unresponsive to medical treatment
Bhasin et al. (2022)	BD-M (27)		+		COVID-19	Olanzapine^**^ 20 mg/day/os; Haloperidol^*^ 20 mg/day/im; Lorazepam^¶^ 2 mg/day/iv	5	Bilateral	Yes (BPRS; YMRS; BFCRS)	Vaginal bleeding; preterm delivery	Transient fetal arrhythmias	ECT is a safe and effective treatment modality in acute psychiatric disorders during second trimester pregnancy where rapidity of response is warranted
Gandhi et al. (2023)	SCZ (25)	+	+			Risperidone^**^ 2 mg/day/os; Paliperidone^**^ 12 mg/day/os; Lorazepam^¶^ 1.5 mg/day/iv	12	N/A	Transient (N/A)	None	None	ECT is recommended in catatonic pregnant patients, especially when symptoms require rapid resolution

^*^ First Generation Antipsychotic drug; ** Second Generation Antipsychotic drug

^†^ Tricyclic Antidepressant drug; †† Selective Serotonin Reuptake Inhibitor

^¶^ Benzodiazepine

*BAI* Beck Anxiety Inventory, *BD-D* bipolar disorder, depressive episode, *BD-M* bipolar disorder, manic episode, *BDI* Beck Depression Inventory, *BFCRS* Bush Francis Catatonia Rating Scale, *BPRS* Brief Psychiatric Rating Scale, *COVID-19* coronavirus disease 2019, *CPZ* chlorpromazine, *Dx* principal diagnosis, *ECT* electro-convulsant therapy, *HAM-D* Hamilton Depression Rating Scale, *MDD* major depressive disorder, *PNX* pneumothorax, *rTMS* repetitive transcranial magnetic stimulation, *SCZ* schizophrenia spectrum disorder, *YMRS* Young Mania Rating Scale, *N/A* not available

Among the low-grade complications in the fetus, a transient alteration of the fetal heart rate was reported in five cases (Varan et al. 1985; DeBattista et al. 2003; Bozkurt et al. 2007; De Asis et al. 2013; Bhasin et al. 2022). This alteration seemed to be without consequent clinical alterations, although all the authors agreed to monitor the fetal heart rate during ECT. O’Reardon reported a case of maternal pneumothorax (moderate-grade complication) that developed during childbirth, with no consequences for the woman and for the child (O’Reardon et al. 2011). Bhasin reported vaginal bleeding and preterm delivery among moderate-grade maternal complications (Bhasin et al. 2022). A preterm birth also occurred in another report (Pinette et al. 2007); however, in this case, there was also pre-eclampsia (moderate-grade complication) and a cerebellar infarction of the unborn child with tonic extension of the upper limbs which could be traced back to ECT-induced uterine contractions (severe-grade complications of the fetus) (Pinette et al. 2007). Balki described a case which ended with severe complications both for the mother (status epilepticus; diabetes insipidus; renal insufficiency; cardiac insufficiency) and for the fetus (abortion) (Balki et al. 2006). Status epilepticus, a rare but known complication of ECT, may have caused both the major complications in the mother and, consequently, fetal death. The prolonged epileptic seizure (201 s) reported by De Asis was not associated with severe or moderate complications in the fetus, but only with brief fetal bradycardia (De Asis et al. 2013); the change of anesthetic agent from methohexital to propofol appears to reduce the risk of prolonged crises.

#### Third trimester of pregnancy

Fourteen case reports were entered into this category (Table 7). Six women were affected by major depressive disorder, six by bipolar disorder (four with current depressive episode, two with current manic episode), and two by schizophrenia. The mean age was 31.2 years, ranging from 22 years (Yellowlees and Page 1990) to 48 years (Salzbrenner et al. 2011). Two women had symptoms of catatonia, eight had psychotic symptoms, and eight had suicidal ideation. Reported comorbidities were as follows: streptococcus B infection, SARS-CoV-2 infection, intellectual disability, arterial hypertension, hypothyroidism, obesity, mellitus and gestational diabetes, alcohol abuse, cocaine abuse, borderline personality disorder, and post-traumatic stress disorder. Pharmacological treatment has frequently been associated with ECT, based on drugs belonging to different classes: first- and second-generation antipsychotics, tricyclic antidepressants, selective serotonin reuptake inhibitors, serotonin and norepinephrine reuptake inhibitors, serotonin reuptake inhibitors and adrenaline, benzodiazepines, and other sedative-hypnotics (Table 7). In nine cases, an improvement in psychiatric symptoms has been reported. In two cases, a temporary improvement was observed, and in one case, a partial response to the treatment was detected (Pesiridou et al. 2010). Notably, the partial response in one report (Pesiridou et al. 2010) and the temporary response in another (Yang et al. 2011) were obtained by the combination of ECT with polypharmacological treatment. In one case (Livingston et al. 1994), a progressive deterioration of the psychiatric condition of the woman has been described even after the execution of ECT, but no test for psychopathological assessment was indicated by the authors. Finally, in only one case (Yellowlees and Page 1990), no information on the clinical outcome of ECT was reported.

**Table 7:** Case reports on women undergoing electro-convulsant therapy during the third trimester of pregnancy

First Author (publication year)	Dx (age)	Mother’s comorbidity				Psychotropic drug(s) taken during ECT	ECT sessions during pregnancy		Symptoms resolution (measure of outcome)	ECT-related complication		Conclusion
		*Catatonic symptoms*	*Psychotic symptoms*	*Suicidality*	*Other*		*n*	*Electrode placement*		*In mother*	*In child*	
Charatan (1954)	SCZ (29)	+	+			None	6	N/A	Temporarily (N/A)	None	None	ECT alone is safe in late-stage pregnancy
Wise (1984)	MDD (24)		+	+	DM	Nortriptyline^†^ 75 mg/day/os	12	Unilateral	Yes (N/A)	Supra-ventricular tachycardia	None	Modern ECT minimizes risk for mother and fetus
Yellowlees and Page (1990)	SCZ (22)	+	+		ID	Amitriptyline^†^	9	Unilateral	Yes (N/A)	None	None	To perform ECT in pregnancy, specialist skills are needed
Sherer et al. (1991)	MDD (35)		+	+		Haloperidol^*^ 20 mg/day/os; Diazepam^¶^ 15 mg/day/os	7	Bilateral	N/A	Vaginal bleeding; Placental abruption	None	Transient hypertension may explain abruptio placentae
Livingston et al. (1994)	MDD (28)		+	+		None	8	Bilateral	**No** (N/A)	Preterm delivery	Death	Twin pregnancy. Anatomical abnormalities detected before ECT; one child dead postpartum due postoperative complications, not ECT
Kasar et al. (2007)	MDD (32)		+	+		Venlafaxine^†††^ 75 mg/day/os; Quetiapine^**^ 300 mg/day/os	4	Bilateral	Yes (HAM-D)	Preterm delivery	None	It is necessary to know and prevent the risks of ECT before treatment
Pesiridou et al. (2010)	BD-D (33)			+	Alc; Cocaine; PTSD; PD-B	Fluoxetine^††^ 40 mg/day/os; Quetiapine^**^ 600 mg/day/os; Lorazepam^¶^ 2 mg/day/os	6	Unilateral	Partial response (BDI; BAI)	Late-onset contraction	None	Uterine contractions may be late in onset, and this is particularly salient when ECT is being conducted on an outpatient. Tocolytics can delay delivery
Salzbrenner et al. (2011)	BD-D (48)			+	HypoT; GD; Ob; AH; StrepB	None	12	Bilateral	Yes (MADRS)	Pre-eclampsia	None	Monitoring of the fetus and mother is essential during ECT
Yang et al. (2011)	BD-D (33)		+			Olanzapine^**^ 7.5 mg/day/os; Risperidone^**^ 3 mg/day/os; Quetiapine^**^ 25 mg/day/os; Trazodone^††††^ 25 mg/day/os; Zolpidem¶¶ 10 mg/day/os	7	N/A	Temporarily (N/A)	Preterm delivery	Hyaline membrane disease; pyloric stenosis	Although ECT typically has little effect on fetal and maternal status, the clinicians should be aware of the complications
Chen (2015)	BD-M (31)		+			None	1	Bilateral	Yes (N/A)	None	None	ECT may be considered in pregnancy, despite its possible complications
Thyen et al. (2017)	BD-M (28)				Cocaine	Risperidone^**^	9	Bilateral	Yes (N/A)	Pre-eclampsia	None	ECT is safe and effective in pregnant women, and it should be considered in appropriate clinical circumstances
Moosavi Rineh et al. (2020)	MDD (26)					None	6	Bilateral	Yes (N/A)	None	Transient fetal arrhythmias	ECT could be a valuable and effective treatment modality in resistance and emergency cases at the late pregnancy
Gannon et al. (2021)	BD-D (33)			+		Quetiapine^**^ 200 mg/day/os; Lurasidone^**^ 90 mg/day/os; Sertraline^††^ 100 mg/day/os	7	Bilateral	Yes (N/A)	Hypomanic symptoms	None	ECT is safe and effective in pregnancy, and it was considered as essential procedure during COVID-19 pandemic
Patel et al. (2022)	MDD (35)			+	COVID-19	Quetiapine^**^ 300 mg/day/os; Fluoxetine^††^ 60 mg/day/os	8	Bilateral	Yes (PHQ-9)	None	Transient fetal arrhythmias	ECT in pregnancy is a viable option to treat TRD, prevent harm to the fetus, and reduce pharmacologic burden

^*^ First Generation Antipsychotic drug; ** Second Generation Antipsychotic drug

^†^ Tricyclic Antidepressant drug; †† Selective Serotonin Reuptake Inhibitor; ††† Serotonin and Noradrenalin Reuptake Inhibitor; †††† Serotonin and Adrenaline Reuptake Inhibitor

^¶^ Benzodiazepine; ¶¶ Other Sedative-Hypnotic agents

**AH**: Arterial Hypertension; **Alc**: Alcohol abuse; **BAI**: Beck Anxiety Inventory; **BD-D**: Bipolar Disorder, depressive episode; **BD-M**: Bipolar Disorder, manic episode; **BDI**: Beck Depression Inventory; **COVID-19**: Coronavirus Disease 2019; **Cocaine**: Cocaine abuse; **DM**: Diabetes Mellitus; **Dx**: Principal diagnosis; **ECT**: Electro-Convulsant Therapy; **GD**: Gestational Diabetes; **HAM-D**: Hamilton Depression Rating Scale; **HypoT**: Hypothyroidism; **ID**: Intellectual Disability; **MADRS**: Montgomery Asberg Depression Rating Scale; **MDD**: Major Depressive Disorder; **Ob**: Obesity; **PHQ-9**: Patient Health Questionnaire-9; **PD-B**: Personality disorder, borderline; **PTSD**: Post-Traumatic Stress Disorder; **SCZ**: schizophrenia spectrum disorder; **StrepB**: Streptococcus B infection; **TRD**: Treatment-Resistant Depression; **N/A:** not available

As regard to complications, Wise (1984) and Gannon et al. (2021) reported only low-grade complications in the mother (supraventricular tachycardia and the onset of hypomanic symptoms, respectively) and there were no complications in the fetus; Moosavi Rineh (Moosavi Rineh et al. 2020) and Patel (Patel et al. 2022) instead reported the appearance of low-grade complications in the fetus (transient fetal arrhythmia in both cases) and no maternal complications. Among the moderate maternal complications, three cases of ECT-induced preterm delivery and two cases of pre-eclampsia were reported (Salzbrenner et al. 2011; Thyen et al. 2017). Sherer et al. (1991) described the onset of vaginal bleeding and placental abruption which can be explained by a transient maternal blood pressure rise induced by the technique, which however did not determine any subsequent consequences for the child. Uterine contractions are a common and well-known complication of ECT that usually occur during the session; however, Pesiridou et al. reported the onset of uterine contractions with threat of abortion which occurred approximately 10 h after treatment (Pesiridou et al. 2010). The possibility of late contractions must be considered especially when treating women as outpatients.

In Yang et al. (2011), pre-term delivery was associated with moderate complications for the child such as hyaline membrane disease and pyloric stenosis that should be not directly related to ECT, although according to the authors it cannot be excluded.

It is of interest the case of a twin pregnancy of a 28-year-old woman with severe major depressive disorder with psychotic symptoms and suicidal ideation (Livingston et al. 1994): after eight ECT sessions were performed, at 35 weeks of pregnancy, the woman was admitted in the hospital for a preterm birth. Both twins had congenital anatomic anomalies, detected before ECT. One female child had anal atresia, small sacral defect, and coarctation of the aorta, while the other child had transposition of the great vessels, which was successfully repaired, but the infant died after surgery. Therefore, this death, which occurred as a result of postoperative complications, cannot be directly linked to the performance of ECT during pregnancy.

### Case series

Eleven studies reporting the cases of more than one woman were placed in this category (Table 8). A total of 96 women were included. In one study (Rabie et al. 2021), information relating to the trimester of pregnancy of five women was not reported; most women (39 out of 96) were in the second trimester of pregnancy, 27 in the first trimester, and 25 in the third trimester. Most women (65 out of 97) were affected by a mood disorder, 16 women by schizophrenia spectrum disorders, and only three women by other psychiatric disorders. The number of ECT sessions, when indicated, was very heterogeneous ranging from a minimum of one session (Ray-Griffith et al. 2016) to a maximum of 23 sessions (Rabie et al. 2021). When reported, the efficacy of ECT was confirmed by these case series (Simon 1948; Laird 1955; Smith 1956; Bhatia et al. 1999; Bulbul et al. 2013; Halmo et al. 2014; Ray-Griffith et al. 2016; Grover et al. 2017; Rabie et al. 2021). Only two studies reported a lack of efficacy of ECT in ameliorating symptoms: ECT failure in 5 out of 33 cases included in Bulbul (Bulbul et al. 2013) and in 11 of 12 cases included in Bulut (Bulut et al. 2013).

**Table 8:** Case series or retrospective studies about women undergoing electro-convulsant therapy during pregnancy

First Author (publication year)	Cases per trimester of pregnancy at the time of ECT			Cases per principal diagnosis/ psychotropic drug(s) taken during ECT			ECT-related complications		Symptoms resolutions (measure of outcome)	Conclusion
	I	II	III	MD	SCZ	Other	In mother	In child		
Simon (1948)	1	2	0	2	–	1	N/A	Death in one case	Yes (N/A)	In one case, which terminated fatally, ECT was delivered seven months before delivery; so probably, it did not cause the infant’s death
				N/A						
Laird (1955)	2	5	1	2	6	–	N/A	None	Yes (N/A)	ECT appeared to be safe during pregnancy
				N/A						
Smith (1956)	3	6	6	12	2	–	N/A	None	Yes (N/A)	No complications or difficulties ensued
				N/A						
Bhatia et al. (1999)	0	0	2	2	–	–-	Uterine contraction	Transient fetal arrhythmias	Yes (N/A)	In the third trimester, it may be prudent to administer ECT in the labor and delivery room under the supervision of an obstetrician
				N/A						
Malhotra (2008)	0	1	1	1	1	–	None	None	N/A	With modern anesthesia techniques and proper team management ECT can be successfully completed
				N/A						
Bulbul et al. (2013)	13	15	5	31	2	–	Uterine contraction	Congenital hip dysplasia; transient heart failure; death in one case	YES In 28/33 cases (CGI-S; HAM-D; YMRS; PANSS)	Physicians should be aware of the potential maternal and fetal complications of ECT
				In 12 patients (36.4%) AD and/or BDZ and/or APS were used						
Bulut et al. (2013)	6	3	3	10	–	2	Pre-term delivery in one case	Club foot deformity in one case	**NO** In 11/12 cases (N/A)	ECT seems to be an effective and safe treatment option for mood disorders of pregnant women
				In 11 patients AD and/or BDZ and/or APS were used						
Halmo et al. (2014)	1	1	1	–	3	–	N/A	Transient fetal arrhythmias; transient fetal spasms	Yes (N/A)	Fetal spasms can occur before maternal movements started; the pathophysiology of the spasms remains unclear. Despite this, ECT did not impact the development of the fetus
				In two cases, APS (Quetiapine 900 mg/day/os) was maintained						
Ray-Griffith et al. (2016)	1	4	3	8	–	–	Hypomanic symptoms; pre-term delivery; complete heart block	None (N/A in some cases)	Yes (N/A)	ECT permits a rapid resolution of maternal symptoms minimizing risk to the fetus particularly in women with suicidality
				N/A						
Grover et al. (2017)	0	2	3	3	2	–	Uterine contraction; pre-eclampsia	None	Yes (N/A)	ECT-induced seizures can cause rise in oxytocin levels, which increases the uterine contractions and induces pre-term labor
				AD and/or MS and/or APS were used						
Rabie et al. (2021)	N/A			5	–	–	Uterine contraction	Transient fetal arrhythmias	Yes (N/A)	ECT is a safe treatment option for mood disorders in the perinatal period. Continuous FHR monitoring can be logistically challenging and do not apport any additional benefit
				In 4 cases AD and/or BDZ and/or MS and/or APS were used						

*AD* antidepressant agents, *APS* antipsychotic agents, *BDZ* benzodiazepines, *CGI-S* Clinical Global Impression Severity, *ECT* electro-convulsant therapy, *FHR* fetal heart rate, *HAM-D* Hamilton Depression Rating Scale, *MD* mood disorder, *MS* mood stabilizer, *PANSS* Positive and Negative Syndrome Scale, *SCZ* schizophrenia spectrum disorder, *YMRS* Young Mania Rating Scale, *N/A* not available

As regard to the complications, uterine contractions were the most common maternal complication (low-grade complication) being reported in four case series (Bhatia et al. 1999; Bulbul et al. 2013; Grover et al. 2017; Rabie et al. 2021). Grover and colleagues reported pre-eclampsia (moderate-grade), and they associated the ECT-induced rise in oxytocin with uterine contractions and the risk of preterm delivery which, however, did not occur in these cases (Grover et al. 2017).

In the case series on six women with mood disorders, Ray-Griffith and colleagues reported only maternal complications of ECT: appearance of hypomanic symptoms (low grade), induction of preterm birth (moderate grade), and complete heart block (severe grade) that, however, did not cause mother and child impairment (Ray-Griffith et al. 2016).

Also, fetal complications have been reported, such as fetal heart rate abnormalities (low-grade complications) (Bhatia et al. 1999; Rabie et al. 2021) and transient fetal spasms that, however, did not have negative consequences on the child (Halmo et al. 2014).

In only three cases, child complications were severe; in two cases, congenital anatomical anomalies such as congenital hip dysplasia (Bulbul et al. 2013) and club foot deformity (Bulut et al. 2013) were observed, which, however, cannot be directly attributed to the ECT technique. Bulbul and colleagues also reported transient heart failure because of a supraventricular tachycardia after myocarditis and infant death (Bulbul et al. 2013).

Finally, Simon in his study of three women suffering from various psychiatric disorders who underwent ECT during pregnancy reported the death of the child in one case; however, it was emphasized that the abortion occurred 7 months after the administration of ECT and therefore cannot be considered a direct cause of the infant’s death (Simon 1948).

## Discussion

Our systematic review collected information from 1947 to 2023 regarding 34 case reports and 11 case series with a total of 130 women treated with ECT during pregnancy. From the data collected, it emerges that ECT is a treatment used in all the stages of pregnancy, more frequently during the second trimester, especially for women with a diagnosis of major depressive disorder, bipolar disorder, and schizophrenia spectrum disorders. In most cases, the choice to practice ECT in pregnancy was due to coexistence of both organic and psychiatric comorbidities, the presence of resistance to other treatments, and the need to stop taking drugs with a teratogenic potential. Notably, all women receiving ECT during the first trimester of pregnancy suffered from a severe form of a psychiatric disorder, with psychotic symptoms or suicidal ideation.

Although most studies did not report criteria and assessment scales for evaluating the efficacy of ECT in ameliorating psychiatric symptoms, the authors of the studies included in the present review showed that ECT had a moderate efficacy in rapidly decreasing the depressive, manic, or psychotic symptoms. In particular, in 70.5% of case reports, an improvement of psychopathological symptoms has been reported, with an ulterior 14.7% with partial or temporary response. These data are confirmed by what emerges from the case series review: In Bulbul, there is an 83% of positive outcome upon 33 women observed (Bulbul et al. 2013), while in Grover, the range of symptom reduction varied from 65 to 81% with two patients of five achieving clinical remission (Grover et al. 2017). These findings confirmed that ECT has similar efficacy in both pregnant and non-pregnant populations, especially among depressed patients (Anderson and Reti 2009). This technique is useful also for a more rapid action than pharmacotherapy in case of critical symptoms, such as suicidal ideation.

From the data collected in the present review, we can assume that ECT performed during pregnancy is not a risk-free procedure. However, the majority of possible complications were low- and moderate-grade and not life-threatening. The most common complication, reported in 10 cases, was fetal arrhythmias which resolved spontaneously without any medical intervention. The event of a preterm delivery has been reported seven times; however, a team made up of gynecologists and obstetricians can intervene quickly without serious consequences. Uterine contractions following ECT were reported in five cases, and in one case, they occurred long after performing the technique. This is a problem especially in the case of outpatients undergoing ECT, but not directly representing a risk for the life of the mother or child. Severe, permanent, or fatal complications were reported 6 times in mothers and 10 times in children representing 15.8% and 43.5% of the complications recorded in the mother and the child, respectively; however, it should be noted that these complications have not been related directly to ECT. Furthermore, it is important to underline that, although these complications are relatively high in numerical terms especially in child populations, they concern a small number of cases as they tend to occur in clusters in the same person (e.g., congenital hip dysplasia, transient heart failure and infant death occurred in the same person in the study of Bulbul et al. (2013)) (Bulbul et al. 2013).

Severe complications have been reported by Balki et al. (2006), Pinette et al. (2007), and De Asis et al. (2013). In the case report of Balki, the mother suffered from status epilepticus, cardiac and renal insufficiency, diabetes insipidus, and the fetus aborted (Balki et al. 2006). Pinette described cerebellar infarct and tonic extension of upper limbs that affected the fetus (Pinette et al. 2007). De Asis also reported a prolonged epileptic crisis in the mother after ECT was performed, without any complication for the child (De Asis et al. 2013). Thus, it is possible that ECT causes prolonged contractions with consequent hypoxic damage in both mother and child, decreasing uteroplacental blood as a result of sympathetic stimulation (Teramo et al. 1979). Following this model, hypoxia may lead to these severe complications reported by the authors. Furthermore, the onset of diabetes insipidus can be due to alteration in releasing of ADH (Senouci et al. 2013). Also, a selective stimulation of prolactin and neurophysin release after ECT is reported (Whalley et al. 1982).

Other severe fetal complications have also been reported by Livingston, Bulbul, and Bulut. However, in all these cases, congenital anomalies were already present in the child before ECT was performed, so they cannot be directly related to the technique (Bulut et al. 2013; Bulbul et al. 2013).

Most of the authors agree in suggesting some measures and precautions to be applied before administering ECT in pregnancy, in order to reduce the risk of complications. First of all, it is essential to provide adequate information to the mother and to collect adequate consent even from family members when the woman cannot provide it, for example, in the case of catatonic symptoms. It could be also useful to carry out the maternal and external fetal monitoring of vital signs during and after ECT treatment (such as blood pressure, heart rate, and blood oxygenation), practicing techniques to limit vena cava compression during II and III trimester of pregnancy (such as adopting a lateral position, lifting women’s right hip with a pillow, manual handling of the uterus, and abundant intravenous hydration), using tocolytics to prevent pre-term delivery, excluding vaginal bleeding or cervical dilatation before performing ECT (Miller 1994). Furthermore, performing ECT with low-risk anesthetics (such as Propofol and Midazolam) (Roberti et al. 2022), in a protected environment and with a trained team, further reduces the risk of serious consequences for both the mother and the child.

## Limitations

Our review has some limitations. First, the absence of clinical studies leads to a series of unavoidable biases, the most important of which is the reporting bias as we tend to report the most extreme cases of clinical experience. Analyzing only case reports and not clinical studies does not allow to weigh the contribution of confounding factors in the onset of complications such as the pharmacological treatment before or during the ECT and the anesthetic used to induce sedation during ECT which may have played a role in some maternal and fetus-related complications.

Secondly, a limited number of case reports have follow-up data so that the late onset of ECT-induced side effects in later life cannot be completely excluded.

Thirdly, the methods used to perform ECT varied over time and also across regions in which ECT was used. Finally, despite the narrow inclusion criteria and the fact that ECT is a psychiatric tool, 19 out 45 selected articles (44.1%) have been published on journals referring to other branches, possibly limiting the completeness of the data related to the assessment of psychopathology, the pharmacotherapy used, as well as the patient’s outcome.

## Conclusions

ECT appears to be an effective practice — the only available strategy in some selected cases such as the presence of severe disorders, treatments resistance, and life-threatening risks for mother and child — to treat severe psychiatric conditions during pregnancy both if applied alone or in combination with pharmacological therapy, even though it is not entirely free from the risk of serious complications, both for mother and child.

However, ECT administration techniques evolved over years, with reference to the machines used, the positioning of the electrodes, the anesthesia performed, and the monitoring of the mother and fetus throughout the duration of the treatment, increasing the degree of safety of this therapeutic strategy and leading us to consider that it appears to be relatively safe and effective during pregnancy. Our findings are in line with previous review works that claim ECT as a safe methodology with low risk of adverse event (Anderson and Reti 2009); all potential risks against benefits should always be considered before proceeding with the treatment, patients should be selected based on clinical and therapeutic needs (Leiknes et al. 2015), and ECT should be performed in the setting of a multidisciplinary care team (Rose et al. 2020).

It would be advisable for researchers to conduct further studies aimed at comparing ECT and pharmacotherapy in terms of risk and benefits balance and developing a correct strategy to reduce risk of ECT-related complications.

## Data Availability

The original contributions presented in the study are included in the article and further inquiries can be directed to the corresponding author.
